# Biochemical investigation of kraft lignin degradation by *Pandoraea* sp. B-6 isolated from bamboo slips

**DOI:** 10.1007/s00449-013-0972-9

**Published:** 2013-07-23

**Authors:** Yan Shi, Liyuan Chai, Chongjian Tang, Zhihui Yang, Yu Zheng, Yuehui Chen, Qingxiu Jing

**Affiliations:** 1 School of Metallurgical Science and Engineering, Central South University, Changsha, 410017 Hunan People’s Republic of China; 2 Chinese National Engineering Research Center for Control and Treatment of Heavy Metal Pollution, Changsha, 410017 People’s Republic of China

**Keywords:** *Pandoraea* sp. B-6, Kraft lignin, Degradation, Black liquor

## Abstract

Kraft lignin (KL) is the major pollutant in black liquor. The bacterial strain *Pandoraea* sp. B-6 was able to degrade KL without any co-substrate under high alkaline conditions. At least 38.2 % of chemical oxygen demand and 41.6 % of color were removed in 7 days at concentrations from 1 to 6 g L^−1^. The optimum pH for KL degradation was 10 and the optimum temperature was 30 °C. The greatest activities of 2,249.2 U L^−1^ for manganese peroxidase and 1,120.6 U L^−1^ for laccase were detected on the third and fifth day at pH 10, respectively. Many small molecules, such as cinnamic acid, ferulic acid, 2-hydroxy benzyl alcohol, and vanillyl methyl ketone, were formed during the period of KL degradation based on GC–MS analysis. These results indicate that this strain has great potential for biotreatment of black liquor.

## Introduction

Lignin is the most abundant aromatic compound on earth and is second only to cellulose in its contribution to living terrestrial biomass [1]. The structural complexity of lignin, its high molecular weight, and its insolubility make its degradation difficult [2]. The biological degradation of lignin is critical to the biospheric carbon and oxygen cycle [3] and is responsible for much of the natural destruction of wood in use. Applications utilizing lignin-degrading organisms or isolated enzymes provide environmentally friendly technologies for the pulp and paper industry and for the treatment of many xenobiotic compounds and dyes [4].

Despite the resistance of lignin to degradation, a number of fungi are able to breakdown lignin. The best characterized degraders are white-rot fungi, in particular *Phanerochaete chrysosporium* and *Phlebia radiata* [5], although brown-rot and soft-rot fungi are also able to degrade lignin. White-rot fungi secrete phenol oxidases, including lignin peroxidase, manganese peroxidase, and laccase, that attack lignin. These enzymes act through radical reactions [6]. Basidiomycetous fungi are the most efficient lignin degraders currently known, but there is as yet no commercial biocatalytic process for lignin depolymerization [7]. Bacteria also have versatile pathways for degradation of aromatic substances, from simple phenols to highly complex lignin and related xenobiotic substances.

Bacterial degradation is also attractive in part because fungal lignin degradation results in the formation of low molecular weight, mostly aromatic carboxylic acids, that may be further metabolized by bacteria [8]. There are literature reports of lignin degradation by a number of bacterial species [9–12]. Recent work also indicates that *Sphingomonas paucimobilis* SYK-6, known to degrade lignin-derived aromatic compounds, degrades lignin [6]. More than 10 lignin-degrading bacteria from several genera of *Serratia*, *Citrobacter*, *Klebsiella*, *Paenibacillus*, *Aneurinibacillus*, and *Bacillus* were isolated from the sludge of pulp paper mill and investigated for the ability to degrade lignin [13, 14] and lignin degradation products [15, 16]. Some of those bacteria have been used in the treatment of black liquor [17–19]. The structure of natural lignin is very complex and intact lignin is not commercially available. Because of similarities to natural lignin, kraft lignin (KL) has been widely used for lignin-related studies. KL is also the major by-product in the relatively high alkaline effluent (black liquor) generated by the pulp and paper industry [20]. Although several bacterial strains had been reported to degrade KL, only a few strains are able to degrade KL from pulp and paper industry waste [17, 18].

The Gram-negative bacterial strain *Pandoraea* sp. B-6 was isolated from eroded bamboo slip steeping fluid derived from Kingdom Wu during the Three-Kingdoms Dynasty of ancient China (A.D. 220–280). *Pandoraea* sp. B-6 can use KL and lignin derivatives as its sole carbon sources under high alkaline conditions. In this study, the abilities of *Pandoraea* sp. B-6 and extracellular ligninolytic enzymes to degrade KL were investigated. The low molecular weight compounds resulted from KL degradation were identified by GC–MS analysis. The partial 16S rRNA gene sequence of the microorganism has been deposited in GenBank with the accession number of JN128829.1. This work indicates that *Pandoraea* sp. B-6 has significant potential for use in lignin degradation and black liquor biotreatment.

## Materials and methods

### Bacterial strain and culture conditions

The bacterial strain *Pandoraea* sp. B-6 was isolated from the steeping fluid of eroding bamboo slips [21]. The bacteria were grown in the Luria–Bertani broth medium at 30 °C with shaking at 120 rpm until the optical density at 600 nm (OD_600_) of inoculum reached approximately 1.0. Two-millimeter aliquots of this culture were aseptically inoculated into triplicate flasks containing 100 ml sterile KL mineral salt medium (3 g KL, 2 g, (NH_4_)_2_SO_4_, 1 g K_2_HPO_4_, 1 g KH_2_PO_4_, 0.2 g MgSO_4_, 0.1 g CaCl_2_, 0.05 g FeSO_4_, 0.02 g MnSO_4_ in 1 litre distilled water, pH 7.0). The flasks were incubated at 30 °C with shaking speed of 120 rpm for 7 days. These cultures were used to investigate the effects of the temperature, pH, and initial KL concentration on KL degradation by *Pandoraea* sp. B-6. The pH values were monitored with a pH meter during adjustment of buffer to required pH.

### Bacterial growth and COD measurements

Degradation experiments were carried out on a rotary shaker (120 rpm) under aerobic conditions at 30 °C, pH 10 for 7 days. The rate of *Pandoraea* sp. B-6 growth was determined by measuring the OD_600_ of cultured samples withdrawn at intervals on a Hitachi U-4100 spectrophotometer using centrifuged uninoculated medium as a control in 1.0-cm cuvette path length cells. The control and cultured samples were centrifuged at 10,000 rpm for 10 min to remove biomass and suspended solids, and the chemical oxygen demand (COD) of the supernatant was measured by the fast digestion-spectrophotometric method (the Environmental Protection Industry Standard of the People’s Republic of China, HJ/T 399-2007).

### Measurement of the color removal

The intensity of color, before and after incubation, was determined by the standard method of the Canadian Pulp and Paper Association [22]. The amount of color present was determined spectrophotometrically and was related to the absorbance of a PtCo standard solution at the same wavelength. The samples were centrifuged at 10,000 rpm for 10 min to remove suspended solids. The pH of the supernatant was then adjusted to 7.6, and thereafter the absorbance at 465 nm against distilled water was measured using the spectrophotometer. The absorbance values (*A*) were then transformed into color units (PtCo) as follows:

$$ {\text{CU }}\left( {\text{PtCo}} \right) = 500 \times {\text{\it A}}_{ 2} /{\text{\it A}}_{ 1} $$

where *A*_1_ corresponds to the* A*_465_ of a 500-CU platinum-cobalt standard solution (0.132); and *A*_2_ is the absorbance of the effluent sample.

### Determination of the ligninolytic enzyme activity

An 1-ml aliquot of culture supernatant, prepared by centrifugation (10,000 rpm for 10 min), was used to determine the activity of laccase, lignin peroxidase, and manganese peroxidase. Laccase activity was determined using 2,2^′^-azino-bis-(3-ethylbenzthiazoline-6-sulphonic acid) (ABTS) as substrate with monitoring at 420 nm (*ε*_420_ = 36,000 mol^−1^ cm^−1^) [23]. Lignin peroxidase activity was determined by the peroxide-dependent oxidation of 2 mM veratryl alcohol to veratraldehyde followed at 310 nm (*ε*_310_ = 9.300 mol^−1^ cm^−1^) [24]. Manganese peroxidase activity was assayed by monitoring the oxidation of 2,6-dimethoxyphenol (2, 6-DMP) to coerulignone at 469 nm (*ε*_469_ = 49,600 mol^−1^ cm^−1^) [25]. The value of *ε* was obtained using the Beer–Lambert law:

$$ { \log }\left( {{\text{\it I}}_{0} /{\text{\it I}}} \right) = \varepsilon {\text{\it Cl}} $$

where *log(I*_*0*_ */I)* is absorbance, *ε* is the proportional coefficient of optical absorption, *C* is the concentration of the sample, and *l* is the optical path length of the cuvette.

### Analysis of small molecule intermediates from KL degradation through GC–MS

Cultured samples of bacteria containing 1 g L^−1^ KL in the medium were periodically withdrawn and centrifuged at 12,000 rpm for 10 min. Supernatants were acidified to pH 2.0 with 6 mmol L^−1^ HCl and then extracted with equal volume of ethyl acetate. Three portions of the extraction were collected, dehydrated over anhydrous Na_2_SO_4_, filtered though filter paper, and evaporated at 40 °C under vacuum on a rotary vacuum evaporator. Then, 0.1 ml dioxane and 0.01 ml pyridine were added to the samples followed by silylation with 0.05 ml trimethylsilyl (TMS). The mixture was heated at 60 °C for 15 min with periodic shaking to dissolve residues. GC–MS analysis of organic extracts residues was conducted using the method reported previously [26]. The identification of low molecular weight compounds as TMS derivatives derived from bacterial degradation was done by comparing their mass spectra with that of the National Institute of Standards and Technology (NIST) library available through the instrument manufacturer and also by comparing the retention time (RT) with those of authentic compounds when available.

## Results and discussion

### Effects of temperature and pH on KL degradation by* Pandoraea* sp. B-6

**Fig. 1:**
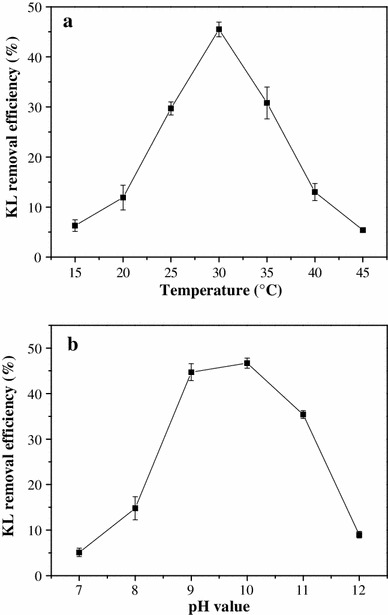
Effect of temperature (**a**) and pH (**b**) on kraft lignin degradation by *Pandoraea* sp. B-6. Data are presented as mean of three replicates with SE

The degradation of KL (3 g L^−1^) by *Pandoraea* sp. B-6 as a function of temperature is shown in Fig. 1a. The extent of KL degradation was significantly different at 15 °C than at 45 °C. The optimum temperature for degradation under these conditions was 30 °C. Approximately, 54.5 % of KL remained after 7 days in the bacterial culture at 30 °C. Degradation efficiency decreased dramatically at both higher and lower temperatures with only 5.4 % degraded after 7 days at 45 °C.

The effect of pH on KL degradation is shown in Fig. 1b. The efficiency of KL (1 g L^−1^) was higher under alkaline conditions (pH 9–11) than at lower pH values. There was no significant difference in the amount of KL degradation at pH 9 vs. 10, with 44.7 and 46.7 % KL remaining after 7 days, respectively. The optimum value was 10.

Treatment of bleached kraft mill effluent (black liquor) with white-rot fungi has been well characterized [17]. The fungi require low pH (4–5) for growth and enzyme activity. The solubility of high molecular weight lignin and derivatives are reduced at low pH compared to alkaline pH, and the pH values of pulp and paper effluent are generally high (10–13). Thus, it would be most economical to develop a degradation method that is effective at alkaline pH. Previously reported optimum pH values for KL lignin degradation by bacterial strains varied from 7 to 8.5. For example, the optimum pH of *Aneurinibacillus aneurinilyticus* was 7.6 [15], that for *Comamonas* sp. B-9 was 7 [26], and that for a *Bacillus* strain was 7.6 [17]. For *Streptomyces* strains, pH optimum ranged from 7.8 to 8.5 [27]. Our results indicate that *Pandoraea* sp. B-6, with its optimum KL degradation activity at high pH, has a significant advantage for application in black liquor biotreatment compared with other microorganisms.

### Bacterial growth and KL degradation

**Fig. 2:**
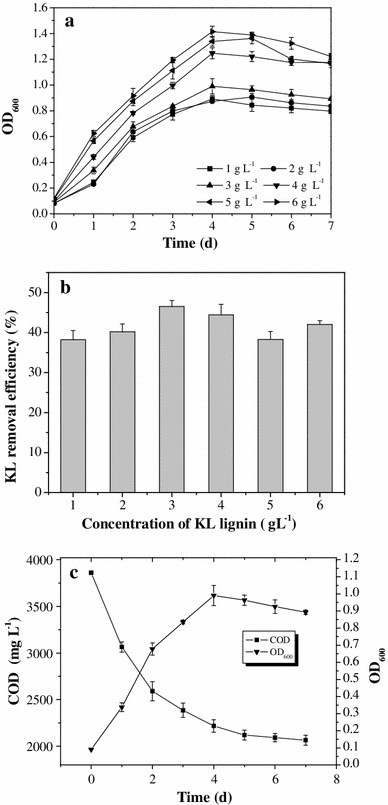
*Pandoraea* sp. B-6 growth and kraft lignin degradation. **a** *Pandoraea* sp. B-6 growth in different initial concentration, **b** kraft lignin removal rate by *Pandoraea* sp. B-6 on day 7 in different initial concentration, and **c** *Pandoraea* sp. B-6 growth and COD reduction in 2 g L^−1^ kraft lignin. Data are presented as mean of three replicates with SE

In order to determine the optimal KL concentration for the degradation reaction, *Pandoraea* sp. B-6 growth was evaluated under seven initial KL concentrations ranging from 1 to 6 g L^−1^. *Pandoraea* sp. B-6 grew fairly well at concentrations from 1 to 6 g L^−1^ (Fig. 2a). The OD value of the cultured sample increased with increasing initial KL concentration. KL degradation at each initial concentration surpassed 38 % after days in culture (Fig. 2b), but there was no obvious correlation between the initial concentration and extent of KL degradation. The highest percent degradation was 46.5 % at the initial KL concentration of 3 g L^−1^; the greatest degradation capacity was 2.52 g L^−1^ for the initial KL concentration of 6 g L^−1^.

The growth of *Pandoraea* sp. B-6 and its ability to degrade KL in the nutrient medium with the KL concentration of 3 g L^−1^ were investigated in detail. The result is shown in Fig. 2c. *Pandoraea* sp. B-6 growth was rapid during the first 2 days in culture and reached the maximum on the fourth day. KL degradation occurred mainly during the first 2 days of culture during the exponential growth phase. Accordingly, the maximum KL removal rate of 795.7 mg L^−1^ day^−1^ was recorded during this period. From the third day, the removal rate decreased gradually, but KL degradation continued. The COD value was 3,860 mg L^−1^ on the first day of culture and was 2,065.1 mg L^−1^ on the seventh day. These results with *Pandoraea* sp. B-6 differ from those of other bacteria such as *Citrobacter* strains. Other strains initially use glucose and peptone as carbon source and subsequently utilize lignin as a co-metabolite [18]. KL can be used as the sole carbon source by *Pandoraea* sp. B-6; accordingly, it was metabolized during the initial growth stage to provide carbon and energy sources. Like KL degradation by *Pandoraea* sp. B-6, *Comamonas* sp. B-9 [26] and *Streptomyces viridosporus* [11] metabolize KL during the initial growth phase and also have a high capacity for KL degradation. These bacterial species metabolize KL throughout the whole life cycle for their growth; therefore, the efficiency and total amount of lignin degradation are higher than with strains that use lignin as a co-metabolite. *Pandoraea* sp. B-6 can be directly used for treatment of black liquor without need for a supplementary carbon source that would increase the COD load of the system.

### KL decolorization

**Fig. 3:**
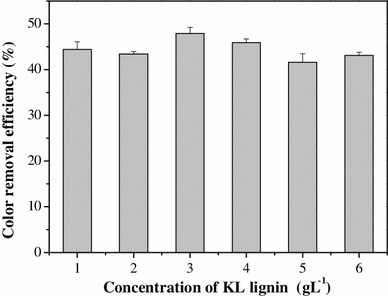
Color removal rate by *Pandoraea* sp. B-6 on day 7 in different initial concentrations of kraft lignin. Data are presented as mean of three replicates with SE

The decolorization of KL at different initial concentrations at pH 10 after 7 days in culture with *Pandoraea* sp. B-6 is shown in Fig. 3. Percent decolorization at initial KL concentrations from 1 to 6 g L^−1^ ranged from 41.6 to 47.9 %. The difference was <7 %, indicating that the effect of initial KL concentration on the *Pandoraea* sp. B-6 decolorization is negligible. The percent decolorization of different initial concentrations of KL at pH 10 over 7 days in culture is shown in Table 1. The decolorization of KL was mainly observed in the first 2 days, consistent with fast bacterium growth and COD reduction. Moreover, like COD reduction, KL decolorization was continued after the second day but at significantly decreased levels. Initially, the color of the culture ranged from 2,651.5 to 18,333.3 CU but dropped to 1,474.2 CU for the lowest KL concentration and to 1,0431.7 CU for the highest KL concentration on day 7. The decolorization rates of some bacterial strain, such as *Bacillus* sp. and *Aneurinibacillus aneurinilyticus* [13, 15] are reportedly more than 50 %. Although these decolorization rates are higher than that of *Pandoraea* sp. B-6, the KL concentrations in cultures of the other strains were <0.6 g L^−1^, much lower than the 3 g L^−1^ KL used in the present study.

**Table 1:** Color removal (change in color units) in six different initial concentration of KL during the process of KL degradation by *Pandoraea* sp. B-6

Time (days)	Different initial concentrations of KL					
	1 g L^−1^	2 g L^−1^	3 g L^−1^	4 g L^−1^	5 g L^−1^	6 g L^−1^
1	2,651.5	5,643.9	8,712.1	11,515.2	15,075.8	18,333.3
2	2,324.8 ± 63.0	4,980.9 ± 82.6	7,783.0 ± 87.4	9,914.6 ± 166.1	13,369.0 ± 268.6	16,267.27 ± 203.7
3	1,801.0 ± 73.5	4,021.8 ± 70.5	6,090.5 ± 62.1	7,951.6 ± 138.1	10,985.5 ± 128.4	12,581.5 ± 171.3
4	1,661.7 ± 47.5	3,549.5 ± 58.1	5,578.3 ± 50.1	7,545.9 ± 126.2	10,207.4 ± 273.5	11,683.4 ± 250.3
5	1,571.0 ± 64.3	3,338.0 ± 54.9	5,339.9 ± 83.4	7,220.3 ± 84.6	9,702.3 ± 133.4	11,484.83 ± 187.1
6	1,498.3 ± 42.5	3,269.5 ± 101.9	4,839.5 ± 61.7	6,398.6 ± 120.6	9,054.8 ± 127.4	11,044.65 ± 257.5
7	1,474.2 ± 55.0	3,194.5 ± 39.8	4,539.0 ± 72.6	6,229.7 ± 100.7	8,804.2 ± 130.4	10,431.7 ± 65.8

### Bioassay of enzymes related to KL degradation

**Fig. 4:**
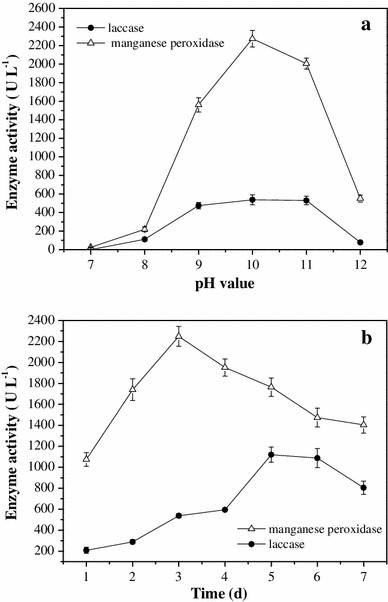
The activity of manganese peroxidase and laccase during the process of kraft lignin degradation by *Pandoraea* sp. B-6. **a** The activity of manganese peroxidase and laccase at different pH values on day 3. **b** The activity of manganese peroxidase and laccase during 7 days at pH 10. Cell-free supernatants were used as enzyme source for ligninolytic enzyme assays. Values are mean of three replicates with SE

Three major extracellular enzymes, including lignin peroxidase, manganese peroxidase, and laccase, carry out lignin degradation and have been well characterized in microorganisms [2]. The activity of these three enzymes from *Pandoraea* sp. B-6 was investigated at different pH values. Results after 3 days in culture of an initial KL concentration of 3 g L^−1^ KL are shown in Fig. 4a. No obvious lignin peroxidase activity was observed at any pH value. The activities of manganese peroxidase and laccase were observed at pH values from 8 to 11, with the maximum values of 2,274 and 537.4 U L^−1^, respectively, at pH 10. The activities of these two enzymes were almost undetectable at pH 7. These results indicate that manganese peroxidase and laccase are basophilic enzymes, although activities were suppressed pH 12.

The activities of manganese peroxidase and laccase during a time course of KL (3 g L^−1^) degradation are shown in Fig. 4b. The manganese peroxidase activity increased significantly during the initial 3 days with the maximum of 2,249.2 U L^−1^ on the third day, decreasing thereafter. The laccase activity was maintained at a low level through the first 2 days. A significant increase in activity was observed on the third day with the maximum of 1,120.6 U L^−1^ on day 5. These results indicate that manganese peroxidase plays a crucial role in KL degradation by *Pandoraea s*p. B-6 beginning on the first day of culture; whereas laccase mainly functions in once the exponential growth phase is almost over. This is similar to the conclusions from previous reports regarding enzymes responsible for KL degradation in other bacterial species [28, 29]. It may be that the secretion of ligninolytic enzymes by *Pandoraea* sp. B-6 occurs mostly during primary metabolism. As *Pandoraea* sp. B-6 is a Gram-negative bacterium, large lignin polymer molecules cannot be passively taken into the cell. Therefore, *Pandoraea* sp. B-6 produces extracellular ligninolytic enzymes to degrade the lignin polymer into fragments that can traverse the bacterial membrane. In addition, no obvious activity of lignin peroxidase was observed during the course of KL degradation, indicating that lignin peroxidase was not produced by *Pandoraea* sp. B-6. Some white-rot fungi and bacteria, such as *Dichomitus squalens*, *Lentinula edodes* [8], and *Comamonas* sp. B-9 [26], that are lignin degraders simultaneously produce manganese peroxidase and laccase, but did not secrete detectable levels of lignin peroxidase. Lignin peroxidase is responsible for the oxidation of non-phenolic syringyl and biphenyl model compounds in certain types of lignin (like hardwood) and subsequent ring cleavage [8]. The mechanism of lignin biodegradation in the absence of lignin peroxidase production is not understood. It is conceivable that the efficiency of hardwood degradation by *Pandoraea* sp. B-6 and the other microorganisms mentioned above is relatively low.

*Pandoraea* sp. B-6 produces extracellular ligninolytic enzymes to degrade the lignin polymer. Activity of ligninolytic enzymes was detected in the cell-free supernatant of bacteria grown at alkaline pH, and the level of manganese peroxidase and laccase was high in alkaline conditions. Unlike most lignin-degrading microorganisms, *Pandoraea* sp. B-6 produced manganese peroxidase and laccase to degrade lignin without need for an exogenous carbon source. We conclude that *Pandoraea* sp. B-6 can be directly used for treatment of black liquor without carbon source supplementation that increases the COD load of the system.

### Metabolite characterization through GC–MS

**Fig. 5:**
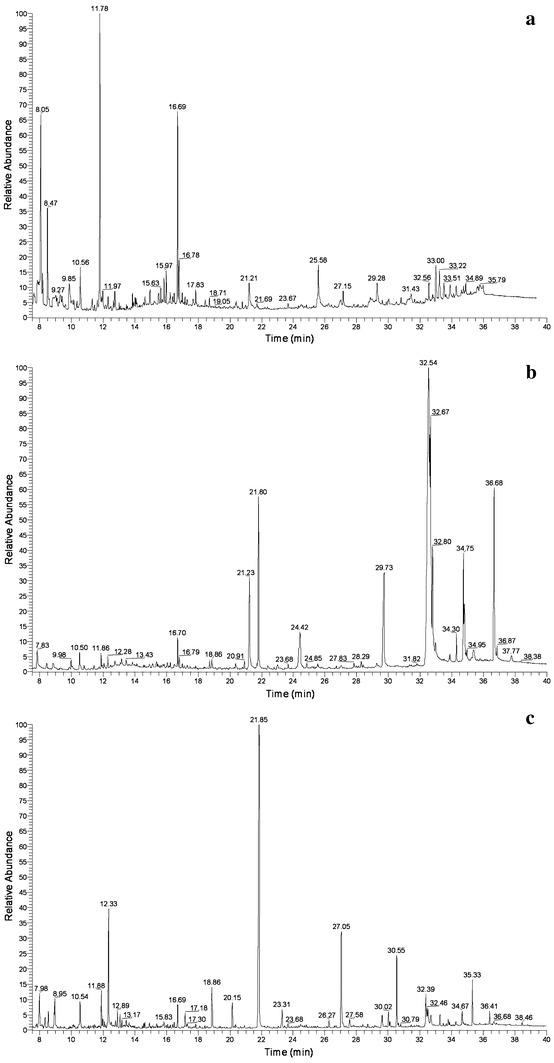
The total ion chromatograph of trimethylsilyl derivatives of compounds extracted with trichloromethane from kraft lignin medium incubated with *Pandoraea* sp. B-6. **a** 0 days; **b** 3 days; and** c** 7 days

The potential of *Pandoraea* sp. B-6 for the treatment of black liquor was further studied by identifying the lignin-related aromatic compounds released during KL degradation using GC–MS analysis. The total ion chromatograph (TIC) patterns corresponding to the compounds extracted with ethyl acetate from the acidified supernatants obtained from the control (uninoculated medium sample) and degraded sample are shown in Fig. 5a, b and their peak identities are listed in Table 2. In the TIC pattern of control sample (Fig. 5a), acetic acid and phenol were identified at retention times (RTs) of 8.05 and 10.56, respectively. These two compounds are the two important intermediate metabolites during the breakdown of lignin by the microorganism [30, 31]. The presence of acetic acid and phenol may be attributed to the chemical oxidation of lignin due to aeration and agitation in the uninoculated culture. Moreover, some other lignin-related compounds were also identified, suggesting that minor degradation of KL occurs during the industrial production process [32].

**Table 2:** Compounds identified as TMS derivatives in chloroform extract from control and bacterial degraded kraft lignin samples as given in Fig. 5

Present in					
No.	RT^a^	Fig. 4a	Fig. 4b	Fig. 4c	Compounds
		(0 days)	(3 days)	(7 days)	
1	8.05	+	–	–	Acetic acid
2	8.47	+	–	–	Methyl acetate
3	10.5	–	+	–	Ethanedioic acid
4	10.56	+	–	–	Phenol
5	11.78	+	–	–	3,5-Dimethyl-4-hydroxybenzaldehyde
6	11.86	–	+	–	Veratryl alcohol
7	12.33	–	–	+	2-Hydroxy-5-methyl-*p*-benzoquinone
8	16.69	–	–	+	Cinnamic acid
9	16.79	–	+	–	Ethylguaiacol
10	18.86	–	+	+	Ferulic acid
11	20.15	–	–	+	4-Hydroxy-3-methoxyphenylacetone
12	21.23	–	+	–	Dibutyl phthalate
13	21.80	–	+	–	4-Hydroxycinnamic acid
14	21.85	–	–	+	Guaiacyl vinyl ketone
15	23.31	–	–	+	3,4,5-Trimethoxy benzaldehyde
16	24.42	–	+	–	3,4,5-Trimethoxycinnamic acid
17	27.05	–	–	+	4-Hydroxy-3,5-dimethoxy benzaldehyde
18	29.73	–	+	–	4-Hydroxy-3-methoxybenzoic acid
19	30.02	–	–	+	2-Hydroxy benzyl alcohol
20	30.55	–	–	+	Hexadecanoic acid
21	32.39	–	–	+	Vanillyl methyl ketone
22	34.75	–	+	–	Lignophenols
23	36.68	–	+	+	3-3′-Dihydroxy-,4,-4′ dimethoxy-6-formyl-6′-methyl-biphenyl carboxylic acid

^a^Retention time

As shown in Fig. 5b, the TIC of a sample of KL inoculated with *Pandoraea* sp. B-6 showed a significant increase in the number of peaks after 3 days of incubation as compared to the uninoculated sample, which indicated that a large number of low molecular compounds were released from the KL polymer as a result of the presence of the bacteria. Furthermore, many peaks identified on day 3 decreased after 7 days of incubation and new peaks appeared, indicating that low molecular weight compounds released form KL polymer initially were further degraded or mineralized. Based on results of the TIC for control and degraded samples, the process of KL degradation by *Pandoraea* sp. B-6 was divided into two steps: the initial depolymerization of KL and the degradation of the lower molecular weight compounds.

Many low molecular weight aromatic compounds were detected in the extract of the degraded sample (Fig. 5b, c; Table 2). These included veratryl alcohol (RT 11.86), cinnamic acid (RT 16.69), ethylguaiacol (RT 16.79), ferulic acid (RT 18.86), 4-hydroxycinnamic acid (RT 21.80), 3,4,5-trimethoxy benzaldehyde (RT 23.31), and lignophenols (RT 34.75), none of which were present in the control sample. The aromatic compounds identified in extract of degraded sample were due to the radical polymerization of guaiacyl (G) units from precursor coniferyl alcohol, syringyl (S) units from precursor sinapyl alcohol, and p-hydroxyphenyl (H) units from precursor p-coumaryl alcohol. G, S, and H units are considered the basic moieties of the lignin structure [30]. Ferulic acid results from formation of ester and ether linkages by reaction of its phenolic group [33]. Cinnamic acid, which is involved in linking the lignin and hemicellulose fraction of lignocellulose, was identified in the extract degraded by *Pandoraea* sp. B-6. During the process of pulping alkaline extraction, most of the ester linkages are broken down, but some cinnamic acid still remains bound to the lignin by ether linkages [32]; these were cleaved by *Pandoraea* sp. B-6. Further, the peak at RT 21.23 was identified as dibutyl phthalate, which reportedly occurs during fungal peroxidase degradation of lignosulfonate [34] and during photo-degradation of black liquor lignin [35]. In addition to aromatic compounds, many acid-type compounds were identified in the control and inoculated sample. As in earlier reports [10, 17, 36], lignin degradation products were more often acid-type compounds than aldehyde and ketone-type molecules. It has been reported that cupric oxide degradation of native lignin from different vegetal tissues yields aldehyde-type compounds in higher abundance than ketones and acid-type compounds [37].

## Conclusion

In this paper, we report in the ability of the *Pandoraea* strain B-6 to efficiently degrade KL. *Pandoraea* sp. B-6 grew well in the sterile KL mineral salt medium without any co-substrate. For KL degradation by *Pandoraea* sp. B-6, the optimum pH was 10 and the optimum temperature was 30 °C. In the range from pH 7 to 12, the greatest COD and color removal and the highest levels of activity of extracellular ligninolytic enzymes (manganese peroxidase and laccase) were observed at pH 10. Many intermediates formed during the process of bacterial degradation of were identified through GC–MS analysis. Our results indicate that *Pandoraea* sp. B-6 has significant potential for use in applications requiring for lignin degradation and for treatment of black liquor or other KL containing pollutants before their release into the environment.
